# A Remarkable Case of Abstinence

**Published:** 1895-12

**Authors:** 


### A REMARKABLE CASE OF ABSTINENCE.

The following case occurred in the practice of Dr. Willan (England) in the year 1795:

"A young man of a studious and melancholic disposition, with a tendency to religious fanaticism, suffering from dyspepsia, decided to abstain from all food. He secluded himself from all friends and relatives, taking only from one-half to one pint of water, flavored with orange-juice, daily, and persisted in this course for fifty-one days. He had undertaken during his fast to copy the Bible in shorthand, which he did up to the second chapter of Kings. During the succeeding ten days to the fifty-first day of fasting his strength failed rapidly; he was no longer able to rise from bed and his friends found him out. He was placed under Dr. Willan on the sixty-first day, who found him terribly emaciated, the abdomen concave from collapse of intestines, and his mind had become imbecile. The following diet was prescribed: One pint of milk for breakfast; one pint of mutton-broth, boiled with barley, for dinner, and one pint of rice-milk for supper.

"For the first few days he improved, and stated that the desire for food, which was very troublesome during the commencement of his fast, ceased after the third day; but on the fifth day from the commencement of the diet prescribed he became sleepless and restless. On the sixth day delirium and mania ensued; pulse 120; hot skin; rigor. Delirium and insomnia continued; pulse smaller and feebler, with increasing emaciation until the eleventh day from commencing food and the seventy-second day from that of abstinence, when he died."

Remarks:

"When the mind is set on the accomplishment of such a fast, especially under the influence of religious mania, it greatly aids the powers of enduring vitality."

"The quantity of food prescribed for a person who had fasted sixty days was excessive and would necessarily preclude all chance of his recovery."

